# Predictors of quality of life among caregivers of patients with moderate to severe kidney disease: an Australian cross-sectional study

**DOI:** 10.1186/s12955-024-02317-z

**Published:** 2024-12-18

**Authors:** Edward Zimbudzi, Asha Blessan, Denise Fraginal, Lelise Gute, Qiumian Wang, Shari Ziganay

**Affiliations:** 1 Department of Nephrology, Monash Health, Melbourne, Australia; 2 Monash Nursing and Midwifery, Monash University, Melbourne, Australia; 3 Chronic and Complex Care Renal Services, Western Health, Melbourne, Australia

## Abstract

**Background:**

Little is known about the quality of life (QoL) of caregivers of patients with chronic kidney disease (CKD) along the disease continuum. We investigated factors associated with low QoL among caregivers of patients with CKD including those on dialysis. We also examined the relationship between kidney disease severity and the QoL of caregivers.

**Methods:**

We recruited caregivers of patients with CKD (stage 3 to 5) attending renal outpatient clinics as well as dialysis units of a tertiary hospital and patients from January 2018 to November 2023. Quality of life was assessed using a valid and reliable tool, the Adult Carer Quality of Life Questionnaire. Logistic regression analyses were performed to determine factors associated with low QoL among caregivers.

**Results:**

A total of 278 dyads of caregivers and patients were studied with a mean age of 56.6 ± 15.2 and 63.7 ± 15.3 years respectively. The proportion of caregivers reporting low to mid-range QoL scores ranged from 37 to 73.3% across the eight domains, with 48% having low to mid-range overall QoL scores. The severity of CKD had no impact on overall QoL of caregivers in the personal growth and carer satisfaction domains where caregivers of patients on dialysis reported worse scores compared to caregivers of predialysis patients. Female gender of caregivers and patients, longer caregiving time, diagnosis of diabetes and lower socioeconomic status of patients were all associated with lower scores in one or more domains.

**Conclusion:**

This study identified several factors associated with low QoL among caregivers of patients with CKD. An understanding of these factors provides insight into the development of targeted interventions to improve the QoL of caregivers.

**Supplementary Information:**

The online version contains supplementary material available at 10.1186/s12955-024-02317-z.

## Background

Previous studies have assessed and reported low quality of life (QoL) among informal caregivers of people with advanced kidney disease including those on dialysis [1–3]. Factors associated with low QoL among caregivers of people with advanced kidney disease include socio-demographic characteristics of caregivers and patients, disease-related factors, caregiving-related factors, environmental factors, and psychological factors [4]. Specifically, some of these factors include caregivers’ age, female gender, ill-health, education, duration of the caregiving role, relationship to patients (spouse or parent), caring for patients receiving haemodialysis, and low socioeconomic status [3–6]. However, current research evidence is inconclusive regarding the influence of other factors such as cultural norms on the QoL of caregivers of people with kidney disease [4].

While a number of factors associated with impaired QoL among caregivers of people with advanced kidney disease are known, there is a knowledge gap regarding the influence of these factors on the QoL of caregivers of people with moderate chronic kidney disease (CKD). Having an insight into the QoL of caregivers for patients in early stages of CKD is of particular interest given the longevity of their caring role along the trajectory of a disease whose severity progresses with time. Additionally, informal caregivers play an important role in the management of CKD, which includes efforts to slow down the progress of the disease in such a way that they often need to change their own lifestyle behaviours to support patients with CKD [7]. Understanding factors that influence the QoL of caregivers in these early stages of CKD is an important step in designing interventions meant to improve their well-being.

Another key knowledge gap is that the relationship between severity of kidney disease and the QoL of caregivers is unknown. While it can be assumed that increasing disease severity would have a negative impact on QoL of caregivers due to increasing care needs, a study among caregivers of people with dementia [8] paradoxically found no association between carer QoL and the severity of the disease. What is known for patients with CKD is that comorbidities compromise the patients’ overall functional and cognitive capacity, thereby increasing caregiver burden [9].

Within this context, there is a need for studies that examine the QoL of caregivers across different stages of CKD. In this study we investigated factors associated with low QoL among caregivers of patients with CKD stages 3–5 including those on dialysis. We also examined the relationship between kidney disease severity and the QoL of caregivers. We hypothesised that kidney disease severity would be associated with low QoL among caregivers of people with moderate to severe CKD.

## Methods

### Study design and participants

This was a cross sectional study of caregivers of patients with CKD attending renal outpatient clinics as well as dialysis units of a tertiary hospital in Victoria, Australia from January 2018 to November 2023. Participants were eligible if (1) they were primary (non-professional and unpaid) caregivers; (2) adults 18 years old or older; (3) they had no obvious cognitive disabilities or language barriers; (4) they were willing to participate in the survey; (5) the person they cared for provided consent to participate and had CKD stages 3 to 5 (eGFR < 60 mL/min/1.73 m^2^) including dialysis. Caregivers of patients with CKD stages 1 to 2 were excluded hence albuminuria or proteinuria was not used in the staging of CKD. Caregivers were conveniently recruited from renal clinics and the in-centre dialysis unit and asked to complete the Adult Carer Quality of Life Questionnaire (AC-QoL) (Appendix 1) after providing informed written consent. Using a margin of error of 5%, confidence interval of 95% and population size of 1000, a sample size of 278 was needed for this cross-sectional study. For each patient who provided consent for the caregiver to be recruited into the study, standardised procedures were used to extract relevant demographic and clinical data from the patient’s medical record. All participants provided written informed consent. Ethics approval was obtained from Monash Health Human Research Ethics Committee.

### Demographic and clinical variables

For patients, data that included age, gender, socio-economic status, smoking, stage of kidney disease, duration of kidney disease, cardiovascular risk factors (hypertension and dyslipidaemia) and diabetes complications were collected from electronic medical records of participating patients. Demographic data was collected as part of the study questionnaire for caregivers. This data included their age, gender, ethnicity, time spend caring per week (in hours) and duration of caring role (in years).

Socio-economic status was estimated using the Australian Bureau of Statistics data [10]. Postcodes were coded according to the Index of Relative Social Disadvantage (IRSD), a composite measure based on selected census variables which include income, educational attainment and employment status. The IRSD scores for each postcode were grouped into quintiles for analysis, where the highest quintile comprises 20% of postcodes with the highest IRSD scores (the most advantaged areas).

CKD stage as defined by the Kidney Disease Outcomes Quality Initiative (KDOQI) was used to define severity of the disease [11]. eGFR was calculated using the CKD Epi formula GFR = 141 X min (Scr/κ, 1) α X max (Scr/κ, 1)-1.209 × 0.993Age X 1.018 × 1.159 where Scr is serum creatinine (mg/dL), κ is 0.7 for females and 0.9 for males, α is − 0.329 for females and − 0.411 for males, min indicates the minimum of Scr/κ or 1, and max indicates the maximum of Scr/κ or 1 [12].

### Outcomes

The QoL of caregivers was assessed using a valid and reliable tool, the Adult Carer Quality of Life Questionnaire (AC-QoL), which is a simple survey that measures QoL in eight separate domains namely: support for caring; caring choice; caring stress; money matters; personal growth; sense of value; ability to care; and carer satisfaction [13]. Scores on the overall questionnaire have a possible range of 0 to 120 with higher scores indicating greater QoL [13]. When categorised into 3, 0–40 indicates a low reported QoL, and may suggest problems or difficulties; 41–80 indicates a mid-range reported QoL and 81 + indicates a high reported QoL. Scores on each of the eight domains have a possible range of 0 to 15, with higher scores indicating greater QoL on that domain [13]. When categorised into 3, 0–5 indicates a low reported QoL, and may suggest problems or difficulties; 6–10 indicates a mid-range reported QoL on that domain and 11 + indicates a high reported QoL on that domain.

### Statistical analysis

First, categorical data is reported as percentages and differences between subgroups analysed using chi-squared tests. Second, continuous data is summarised as means with standard deviations and subgroup analysis performed by a two-tailed t-test when data is normally distributed and one-way ANOVA when there are three or more groups. Third, scores of caregivers for patients who had not started dialysis and those who were on dialysis were compared using Wilcoxon-Mann-Whitney test. Fourth, to determine factors associated with QoL of caregivers for patients with CKD, crude and adjusted analyses of the eight domains of the AC-QoL were performed using multivariable logistic regression methods. Due to small numbers of participants reporting low QoL in the eight domains and the overall score, low to mid-range scores were combined to indicate impaired QoL and used as the dependent variable. All relevant independent variables regardless of their univariate results, were included in the multivariate logistic regression models because they were clinically important and warranted inclusion despite their statistical performance. The study had few independent variables, which ensured that the models were parsimonious and had improved generalisability beyond the current study. Confidence intervals were reported at the 95% level and for all analyses, a two-sided significance level of < 0.05 was considered statistically significant. Cases with missing values were included in the analyses after checking for the amount of missing data which was minimal (less than 1%) for variables analysed. Data was missing completely at random. All analyses were performed with Stata V.17.1 (StataCorp).

## Results

### Demographic and clinical characteristics of participants

**Fig. 1:**
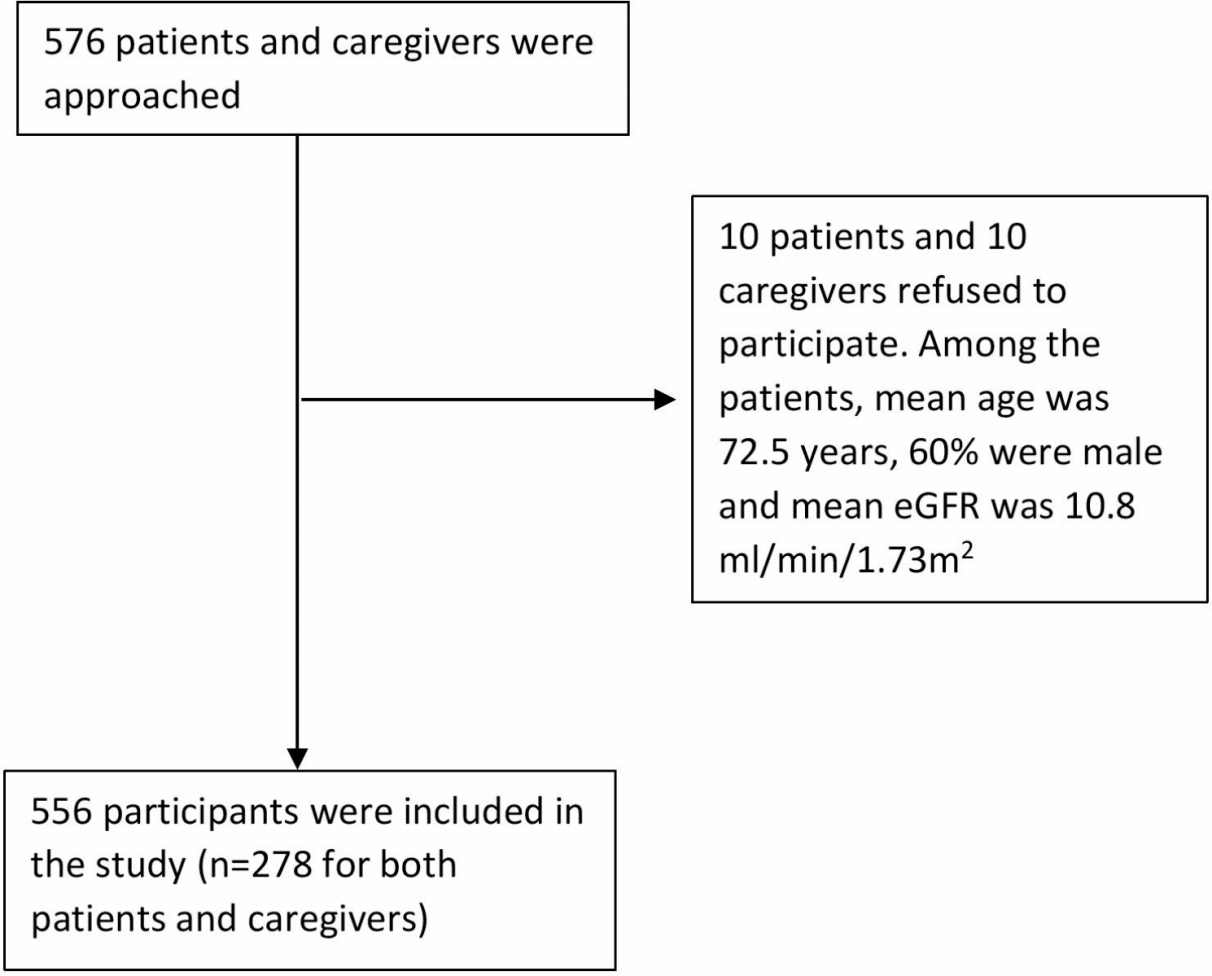
Participant recruitment

Of the 576 patients and caregivers who were invited to participate, 20 refused (Fig. 1). The demographic and clinical characteristics of the 278 dyads of caregivers and patients with CKD who were included in the study are shown in Table 1. The mean age (standard deviation) of caregivers was 56.6 (15.2) years, 34.6% were male and 45.6% identified themselves as white. Approximately, 50% of caregivers spent over 61 h per week providing care and 67% had been in a caring role for less than 5 years. The mean age (standard deviation) for patients was 63.7 ± 15.3 years, 58.6% were male and 63.4% were from culturally and linguistically diverse backgrounds (CALD). Over 60% of patients were on dialysis, 35.7% were classified as having severe comorbidity and 55% had diabetes.

**Table 1:** Participant characteristics

	Mean ± SD or *N* (%)
Care givers	
Age (years)	56.6 ± 15.2
Gender (male)	92 (34.6)
Ethnicity (N, %)	
White	120 (45.6)
Mixed	11 (4.2)
Other	132 (50.2)
Time spend caring per week (in hours)	
0–30	91 (39.1)
31–60	31 (13.3)
Over 61	111 (47.6)
Duration of caring role (years)	
0–5	170 (67.2)
6–10	42 (16.6)
11–15	14 (5.5)
16–20	13 (5.1)
Over 21	14 (5.5)
Quality of life scores (range 0–15)	
Domain 1: Support for Caring	8.8 ± 4.2
Domain 2: Caring Choice	9.9 ± 4.5
Domain 3: Caring Stress	10.7 ± 3.9
Domain 4: Money Matters	8.1 ± 3.8
Domain 5: Personal Growth	9.3 ± 3.9
Domain 6: Sense of Value	11.7 ± 3.6
Domain 7: Ability to Care	11.0 ± 3.3
Domain 8: Carer Satisfaction	11.2 ± 2.9
Overall quality of life score (range 0-120)	80.7 ± 19.2
**Patients**	
Age (years)	63.7 ± 15.3
Gender (male)	163 (58.6)
CALD status (Yes)	173 (63.4)
Stage of kidney disease	
3	14 (5.4)
4	52 (18.7)
5	212 (76.3)
Dialysis modality (N, %)	
Hemodialysis	154 (55.4)
Peritoneal dialysis	19 (6.8)
Predialysis	105 (37.8)
Socio-economic status (quintiles)	
Upper	45 (16.4)
Upper middle	79 (28.7)
Lower middle	52 (18.9)
Upper lower	36 (13.1)
Lower	63 (22.9)
Charlson Comorbidity Index	
Mild (1–2)	72 (26.5)
Moderate (3–4)	103 (37.9)
Severe (greater or equal to 5)	97 (35.7)
Diabetes (Yes)	150 (55.0)

SD-standard deviation, CALD-culturally and linguistically diverse background

### Quality of life scores

**Fig. 2:**
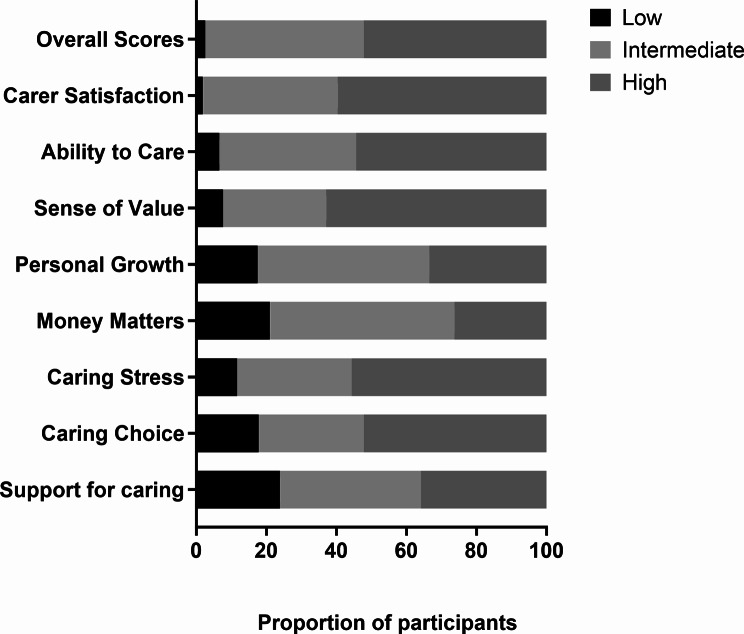
Number and proportion of participants in respective quality of life domains

The mean QoL scores for caregivers across the 8 domains ranged from 8.1 to 11.7 and the overall QoL score was 80.7 ± 19.2, range 11 to 120 (Table 1). The proportion of caregivers reporting low to mid-range scores ranged from 37 to 73.3% across the 8 domains with at least one in five participants reporting low scores in the money matters and support for caring domains, and 48% having low to mid-range overall QoL scores (Fig. 2).

A subgroup analysis (Table 2) showed that younger caregivers (< 65 years) had lower scores in the support for caring [mean difference (MD) -1.15, 95% CI − 2.24 to − 0.07, *p* = 0.04] and money matters domain (MD-1.32, 95% CI − 2.30 to − 0.33, *p* = 0.01) and those who reported longer duration of caring (over 61 h per week) had lower scores in the caring choice, caring stress and money matters domains (all *p* < 0.05). Caregivers of younger patients reported lower scores in the money matters domain (MD -1.09, 95% CI − 1.98 to − 0.20, *p* = 0.02) and those who cared for patients with severe comorbidity reported lower scores in the caring choice, caring stress, personal growth and ability to care domains (all *p* < 0.05). Caregivers of patients with diabetes reported lower scores in the caring stress domain (MD -1.37, 95% CI 0.43 to 2.31, *p* < 0.001) and the overall QoL score (MD -4.97, 95% CI 0.44 to 9.50, *p* = 0.03).

**Table 2:** Comparison of caregiver and patient factors using independent samples t-test and one-way ANOVA

	Mean quality of life scores (SD)								
Characteristic	Support for caring	Caring choice	Caring stress	Money matters	Personal growth	Sense of value	Ability to care	Carer satisfaction	Overall scores
Care givers									
Age									
<65 years	8.4 (4.1)	10.1 (4.4)	10.8 (3.6)	7.7 (3.7)	9.3 (3.9)	11.6 (3.7)	10.7 (3.3)	11.0 (2.9)	79.7 (20.0)
≥65 years	9.6 (4.3) *	9.4 (4.8)	10.4 (4.6)	9.0 (3.9) **	9.2 (4.1)	11.8 (3.6)	11.6 (3.3)	11.5 (2.8)	82.7 (19.2)
Gender									
Male	8.8 (4.6)	9.9 (4.2)	10.9 (3.7)	8.3 (3.7)	8.7 (4.2)	12.1 (3.5)	10.6 (3.4)	11.0 (2.8)	80.6 (17.3)
Female	8.7 (4.0)	9.8 (4.7)	10.6 (4.1)	8.0 (0.5)	9.5 (3.7)	11.5 (3.7)	11.2 (3.3)	11.3 (2.9)	80.8 (20.0))
Caring time									
0–30 h	9.6 (3.8)	11.3 (3.8)	12.0 (3.3)	8.9 (3.9)	9.0 (4.0)	11.8 (3.6)	10.7 (3.3)	11.0 (3.1)	84.2 (19.1)
31–60 h	8.4 (3.7)	8.9 (3.9)	10.0 (3.4)	7.3 (3.3)	9.3 (3.5)	11.9 (3.9)	11.7 (3.6)	11.9 (2.1)	79.4 (16.4)
Over 61 h	8.6 (4.6)	8.8 (5.0) ***	9.8 (4.6) ***	7.5 (3.9) *	9.9 (3.8)	11.8 (3.7)	11.4 (3.5)	11.5 (2.5)	79.3 (20.5)
**Patients**									
Age									
<65 years	8.4 (4.3)	10.1 (4.5)	10.9 (3.6)	7.6 (3.7)	9.5 (3.8)	11.5 (3.6)	10.9 (3.3)	11.1 (2.9)	80.3 (18.6)
≥65 years	9.1 (4.1)	9.8 (4.5)	10.6 (4.3)	8.7 (3.8) *	9.1 (4.0)	12.0 (3.5)	11.0 (3.4)	11.3 (2.9)	81.1 (19.7)
Gender									
Male	10.9 (3.0)	9.9 (4.4)	10.6 (3.9)	7.9 (3.7)	8.8 (4.0)	11.6 (3.5)	11.9 (3.7)	10.9 (3.0)	79.5 (18.3)
Female	11.4 (2.8)	10.0 (4.5)	10.9 (3.9)	8.2 (3.9)	9.6 (3.8)	11.9 (3.7)	11.6 (3.5)	11.4 (2.8)	81.6 (19.7)
Dialysis									
HD	8.6 (4.4)	9.8 (4.5)	10.6 (4.1)	8.0 (3.8)	9.6 (4.0)	12.0 (3.5)	11.2 (3.3)	11.4 (2.9)	81.4 (20.0)
PD	9.8 (3.6)	8.3 (5.4)	10.3 (4.0)	8.6 (4.4)	9.6 (3.9)	11.6 (4.8)	11.4 (3.5)	11.7 (3.7)	80.7 (21.4)
Predialysis	8.8 (3.9)	10.5 (4.2)	11.1 (3.7)	8.3 (3.7)	8.7 (3.7)	11.3 (3.5)	10.6 (3.2)	10.8 (2.6)	79.7 (17.9)
CCI									
Mild	9.1 (4.0)	10.5 (4.4)	11.5 (3.7)	8.1 (4.1)	9.9 (3.8)	12.4 (3.7)	11.6 (3.1)	11.7 (2.9)	83.6 (21.3)
Moderate	8.4 (4.0)	10.9 (3.7)	11.4 (3.3)	8.6 (3.7)	8.4 (3.9)	11.4 (3.5)	10.4 (3.2)	11.0 (2.7)	80.4 (15.7)
Severe	8.9 (4.4)	8.4 (4.9) ***	9.3 (4.4) ***	7.6 (3.6)	9.7 (3.8) *	11.5 (3.5)	11.2 (3.4) *	11.1 (2.9)	78.0 (19.9)
Diabetes									
Yes	8.5 (4.1)	9.5 (4.7)	10.1 (4.1)	7.5 (3.7)	9.0 (4.0)	11.5 (3.6)	10.9 (3.3)	11.1 (2.7)	78.0 (17.2)
No	9.0 (4.1)	10.3 (4.2)	11.5 (3.6) ***	8.9 (3.8) ***	9.5 (3.7)	12.0 (3.6)	11.1 (3.3)	11.3 (3.1)	83.0 (20.7) *

SD-standard deviation; * *p* < 0.05; **p < 0.01; ***p < 0.001; SD-standard deviation; HD-hemodialysis; PD-peritoneal dialysis; CCI-comorbidity index

**Fig. 3:**
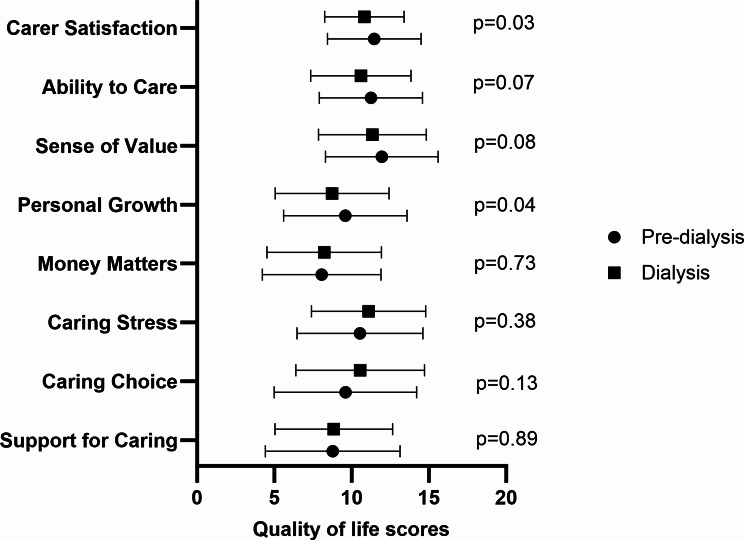
Quality of life scores by disease severity

Caregivers of patients who were on dialysis had significantly lower QoL scores in the personal growth (*p* = 0.04) and carer satisfaction (*p* = 0.03) domains compared to those who cared for pre-dialysis patients (Fig. 3). The overall QoL score was not different between caregivers of patients who were on dialysis and those who were not.

### Caregiver factors

**Fig. 4:**
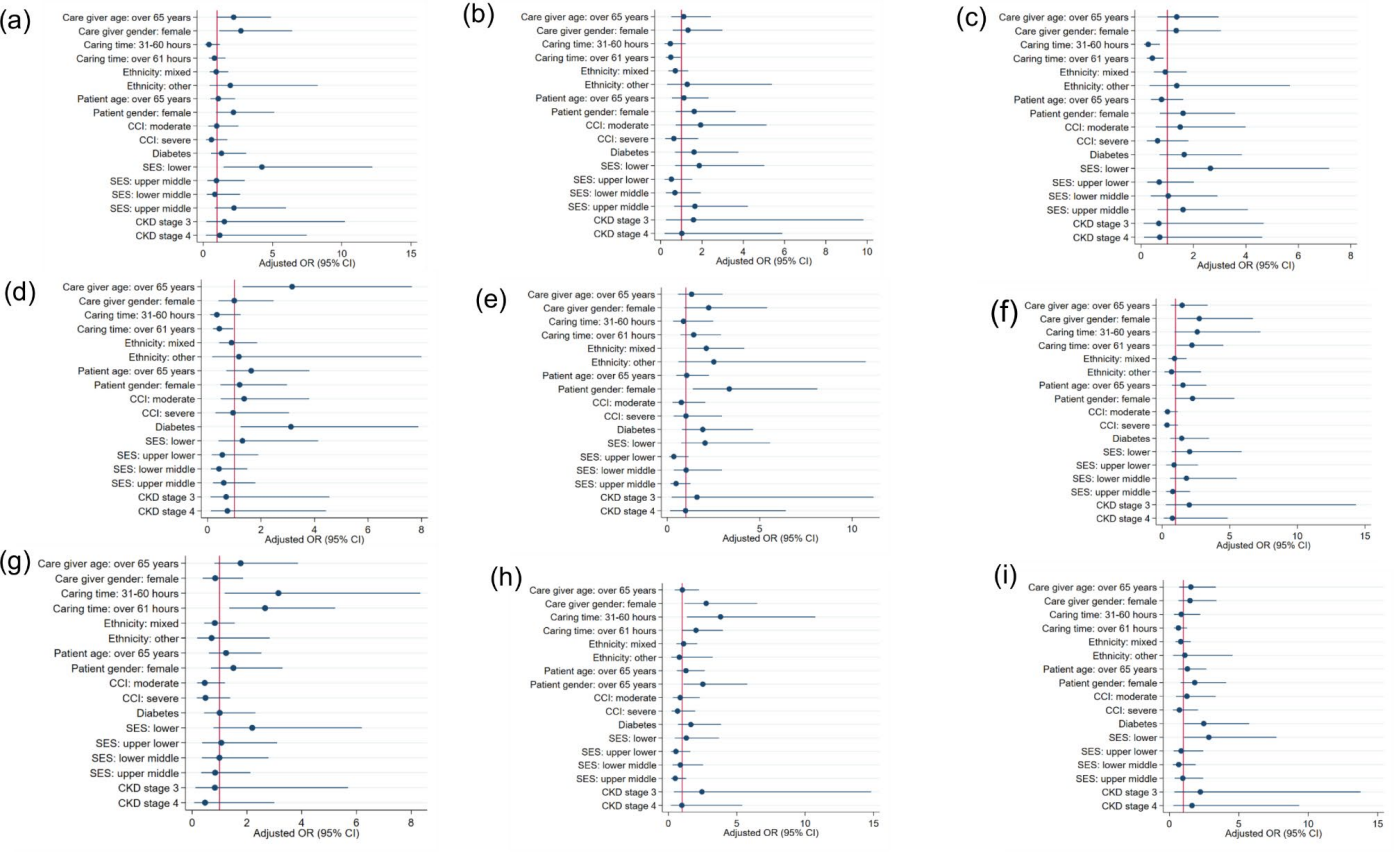
Logistic regression for (**a**) Support for caring, (**b**) Caring choice, (**c**) Caring stress, (**d**) Money matters, (**e**) Personal growth, (**f**) Sense of value, (**g**) Ability to care, (**h**) Carer satisfaction and (**i**) Overall score. References: age < 65 years, gender –male, Caring time-<30 h per week, ethnicity-white, comorbidity index (CCI)-mild, diabetes-without diabetes, socioeconomic status (SES)-upper, chronic kidney disease (CKD)- stage 5

Factors associated with low QoL among caregivers based on the multivariable logistic regression models are shown in Supplementary Tables 1 and Fig. 4. Older age among caregivers was associated with increased odds of low to mid-range scores in the money matters domain [Odds Ratio (OR) 3.16; 95% Confidence Interval (CI) 1.31 to 7.63, *p* = 0.01]. Female gender was associated with increased odds of low to mid-range scores in the support for caring (OR 2.72; 95% CI 0.99 to 4.88, *p* = 0.02), sense of value (OR 2.76; 95% CI 1.13 to 6.72, *p* = 0.03) and carer satisfaction domains (OR 2.76; 95% CI 1.17 to 6.49, *p* = 0.02). Longer caregiving time (over 61 h a week) was associated with lower odds of low to mid-range scores in the caring stress (OR 0.43; 95% CI 0.22 to 0.85, *p* = 0.02) and money matters domains (OR 0.44; 95% CI 0.20 to 0.97, *p* = 0.04) and increased odds of low to mid-range scores in the sense of value (OR 2.21; 95% CI 1.08 to 4.53, *p* = 0.03), ability to care (OR 2.66; 95% CI 1.36 to 5.22, *p* < 0.01) and carer satisfaction (OR 2.01; 95% CI 1.02 to 3.98, *p* = 0.04) domains.

### Patient factors

Female gender among patients was associated with increased odds of low to mid-range scores in the personal growth (OR 3.35; 95% CI 1.38 to 8.12, *p* < 0.01) and carer satisfaction (OR 2.52; 95% CI 1.09 to 5.78, *p* = 0.03) domains (Supplementary Tables 1 and Fig. 4). A diagnosis of diabetes was associated with increased odds of low to mid-range scores in the money matters domain (OR 3.12; 95% CI 1.23 to 7.88, *p* = 0.02) and overall QoL score (OR 2.48; 95% CI 1.07 to 5.75, *p* = 0.04) and lower socioeconomic status was associated with increased odds of low to mid-range scores in the support for caring (OR 4.23; 95% CI 1.46 to 12.21, *p* = 0.01) and overall QoL score (OR 2.84; 95% CI 1.05 to 7.71, *p* = 0.04) (Supplementary Tables 1 and Fig. 4).

## Discussion

In this cross-sectional study, we assessed QoL and associated factors in a sample of 278 dyads of caregivers and patients with CKD. Kidney disease severity had a significant impact on QoL of caregivers in the personal growth and carer satisfaction domains where caregivers of patients on dialysis reported worse scores compared to caregivers of predialysis patients. Female gender of caregivers and patients, longer caregiving time, diagnosis of diabetes and lower socioeconomic status of patients were all associated with lower scores in one or more domains.

These findings contribute to previous research on caregivers of people with ESKD including those on dialysis [1, 2, 6, 14] by characterising and exploring factors that are independently associated with the QoL of caregivers. Most importantly, unlike previous research, this study examines the QoL of caregivers of patients with moderate to severe kidney disease using a measure specific to caregivers. In this study, caregivers exhibited superior QoL scores in comparison to caregivers of patients with cancer [15] and dementia [16].

We show that female gender of caregivers and patients with CKD are associated with low caregiver scores in several QoL domains. This is important because most of the caregivers in our study and other studies in people with ESKD are females [17]. This finding is consistent with previous reports among people with ESKD including those on dialysis [3, 18]. A possible explanation for this finding is that gender differences and burdens may stem from traditional gender roles, as women are often viewed as the primary caregivers in social and informal care settings [19]. Additionally, besides providing care to patients, women tend to have other competing care demands that include providing care to children and their families. We suggest gender-specific support for female caregivers who seem to be particularly vulnerable. While the quality of relationships between caregivers and the people they care for significantly affects caregiving burden and caregiver satisfaction, we did not collect data on these relationships in this study [20]. However, it remains important to explore caregiver dynamics, as spousal relationships can offer protective benefits [21].

Several studies among caregivers of patients with ESKD [22, 23] and other chronic conditions [24] have shown that a longer caregiving time is associated with impaired QoL. While our findings support previous studies, we have gone a step further by exploring the influence of caregiving time on different domains of QoL as measured by the Adult Carer Quality of Life Questionnaire. We report that longer caregiving time was protective of QoL in the caring stress and money matters domains. A possible explanation for this is that caregivers who spent longer caregiving time may not have other competing needs such work commitments and they are potentially well-supported in this role. We have also observed an association between longer caregiving time and low QoL in the sense of value, ability to care and carer satisfaction domains. A probable intervention to improve QoL in these domains is to formalise caregiver education. A similar approach is an intervention which provides education on psychosocial support, symptom management and resource identification for caregivers of patients with cancer [25]. Caregivers involved in the program reported improved perceptions of their health even with increasing caregiving tasks.

Previous studies among caregivers of patients with diabetes only [26, 27] have reported impaired QoL. Reasons for this include that patients with diabetes often have complications, which can have significant impact on the social functioning of caregivers. While there is very limited research on the QoL of caregivers of people with comorbid diabetes and CKD, our data demonstrates that caregiving for people with co-existing diabetes and CKD is associated with worse QoL. This can be explained by the fact that care coordination and distress increase in case of multimorbidity. Integrated services can be set up to reduce the care burden experienced by caregivers of patients with comorbid diabetes and CKD. In a previous study [28], we have demonstrated that such a service results in better integration of care and a perception of improved health and management of health.

Our data also shows that lower socioeconomic status of patients with CKD is associated with low self-reported QoL scores in the support for caring domain and overall scores among caregivers. While we did not specifically collect socioeconomic data for caregivers, it is most likely that socioeconomic status of patients and caregivers was similar. Acknowledging this assumption, these findings are consistent with previous studies among caregivers of patients with cancer [29, 30], adults with ESKD [4] and children on peritoneal dialysis [31], which report that caregivers with higher socioeconomic resources in terms of wealth generally experience higher life satisfaction. These findings raise important questions regarding how the socioeconomic barrier of both patients and caregivers can be bridged to improve the QoL of caregivers.

While we hypothesised that kidney disease severity would be associated with low QoL among caregivers of people with moderate to severe CKD, our study showed that disease trajectory does not significantly impact caregivers’ overall QoL, contrary to common belief. We suggest that this could be due to the distress associated with the diagnosis of CKD that has been previously reported [32]. Additionally, caregivers of newly diagnosed patients may face adjustment challenges related to relational concerns and social support [33]. This underscores the importance of providing support to caregivers, even in the early stages of CKD. Ensuring caregivers have the resources and assistance they need can significantly impact their well-being and the care they provide.

Our findings have important practice and research implications. First, we have identified subgroups of caregivers who may benefit from interventions designed to improve QoL. Psychosocial interventions such as stress management skills, counselling, mindfulness and gratitude training [34–36] should be offered to subgroups of caregivers with low QoL identified in our study. Practical support options such as linkage with support networks and community services, respite care and financial support should also be explored where possible to better support these subgroups of caregivers [36]. Second, future research needs to incorporate the perspectives of people with the lived experience of caregiving for patients with CKD. This brings in new insights into the codesigning of interventions that may lead to optimal QoL among caregivers.

Our data should be interpreted in light of the strengths and limitations associated with our study design. Limitations include the use of data that is based on self-reports of caregivers’ perspectives regarding their QoL. The AC-QoL was completed mostly in the presence of patients and in view of this caregivers may have provided socially acceptable responses especially to questions that inferred on their relationships with patients resulting in overestimation of their QoL. Additionally, patients with advanced stages of CKD were over represented even though we aimed to recruit patients with eGFR < 60 mL/min/1.73m^2^. Another apparent limitation was the exclusion of specific subgroups of participants, such as caregivers of people with CKD stages 3–4 in the community, individuals from rural or regional areas, and those who were not fluent in English. While the AC-QoL has been previously used in caregivers of patients receiving haemodialysis [37], it has not been validated in Australia. We also acknowledge the problems inherent in using an area-based measure as an indicator of socio-economic background, particularly with a relatively small sample size. We collected only a limited number of caregiver-related variables for the analysis, which restricted our ability to explore other important factors, such as caregivers’ health status, previous mental health diagnoses, and additional support. Lastly, the cross-sectional design did not allow us to establish causality between explanatory variables and the QoL of caregivers. To address this, longitudinal studies are needed to have a better understanding of factors that influence QoL in caregivers of patients with moderate to severe CKD. The strengths include the inclusion of several patient and caregiver variables as potential factors that influence QoL of caregivers.

In conclusion, among caregivers of patients with CKD, female gender of both caregivers and patients, longer caregiving time, lower socioeconomic status and a diagnosis of diabetes among patients were independently associated with low QoL. An understanding of these factors provides insight into the development of targeted interventions to improve QoL of caregivers and outcomes of patients with CKD.

## Electronic supplementary material

Below is the link to the electronic supplementary material.

Supplementary Material 1: Appendix: adult Carer Quality of Life Questionnaire

Supplementary Material 2: Table S1: Multiple logistic regression models showing factors associated with caregiver quality of life.

Supplementary Material 3

## Data Availability

No datasets were generated or analysed during the current study.
