# Supplementary material for: Predictors of quality of life among caregivers of patients with moderate to severe kidney disease: an Australian cross-sectional study
Source: Health Qual Life Outcomes. 2024 Dec 18;22:106. doi: 10.1186/s12955-024-02317-z (PMC11657763; doi:10.1186/s12955-024-02317-z)

Manual for the Adult Carer Quality of Life Questionnaire (AC-QoL)

**Hannah Elwick, Stephen Joseph,
Saul Becker & Fiona Becker**

This manual is intended to provide researchers and practitioners in the field of caring with a relevant and accessible instrument to assess the quality of life of adult, unpaid carers. The questionnaire can be used on a one off basis for the purpose of assessment, or as a pre and post intervention tool to measure change and the impact of support.

This manual will be of interest to professionals in the fields of health and social care as well as those researching care-giving and caring organisations themselves.

Published by The Princess Royal Trust for Carers in association with the School of Sociology and Social Policy, The University of Nottingham.

ISBN: 13 9780853582748

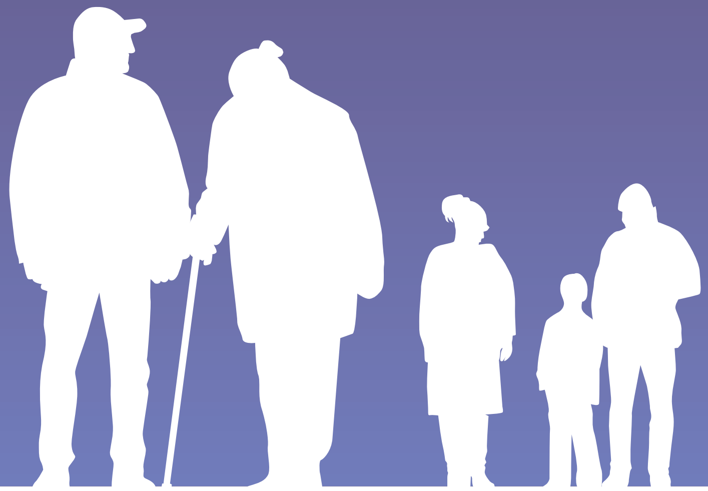

The Princess Royal Trust *for Carers*

The University of Nottingham

Manual for the Adult Carer Quality of Life Questionnaire (AC-QoL)

Hannah Elwick, Stephen Joseph,
Saul Becker & Fiona Becker

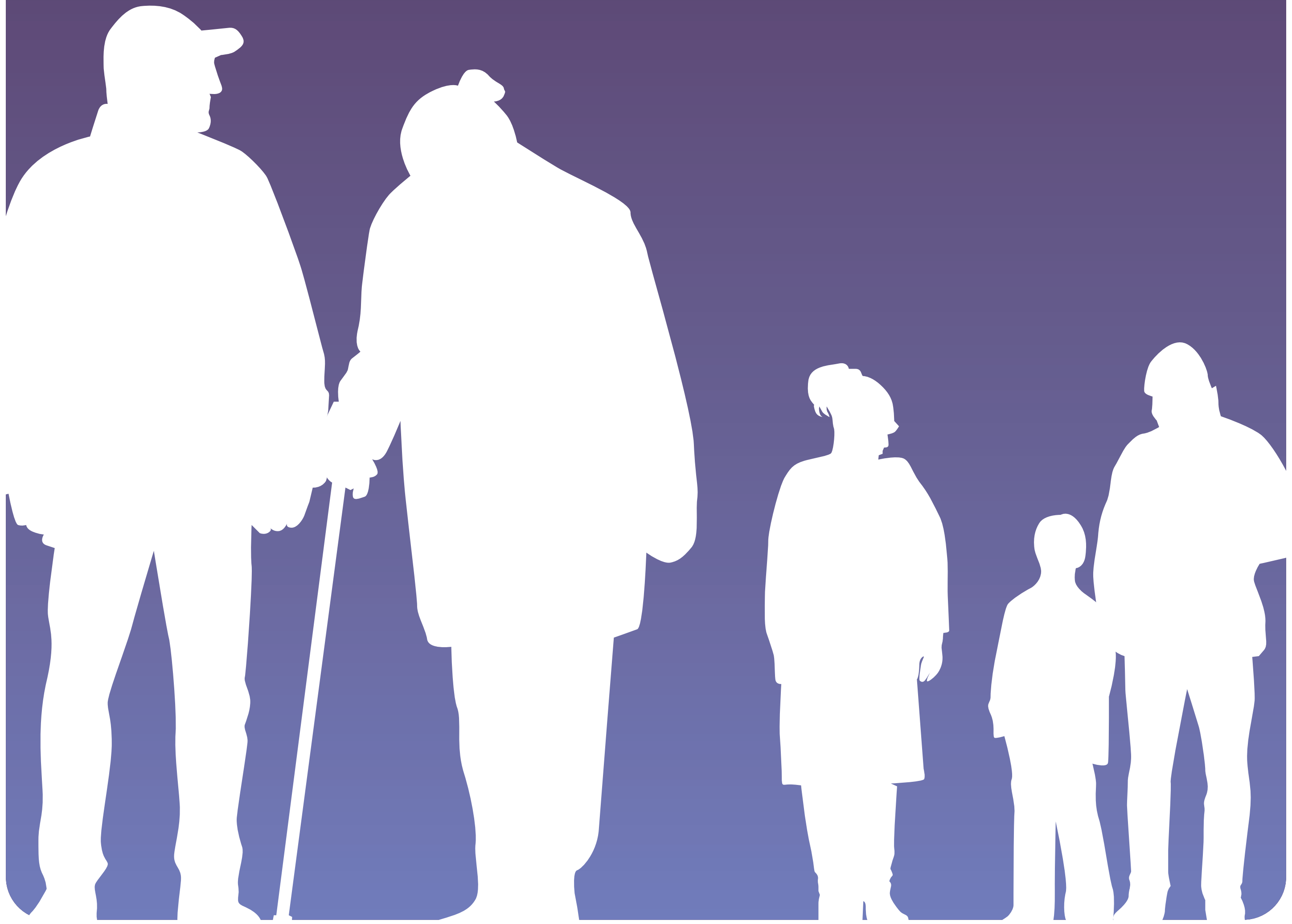

The Princess Royal Trust *for Carers*

The University of Nottingham

Manual for the

Adult Carer Quality of Life Questionnaire (AC-QoL)

Hannah Elwick, Stephen Joseph,
Saul Becker & Fiona Becker

The Princess Royal Trust for Carers

The University of Nottingham

First published in Great Britain in November 2010 by The Princess Royal Trust for Carers, Unit 14, Bourne Court, Southend Road, Woodford Green, Essex, IG8 8HD, in association with The University of Nottingham, School of Sociology and Social Policy, University Park, Nottingham, NG7 1RD.

© Hannah Elwick, Stephen Joseph, Saul Becker, Fiona Becker

Hannah Elwick is a Research Fellow in the School of Sociology and Social Policy, The University of Nottingham.

Stephen Joseph is Professor of Psychology, Health and Social Care, and Director, Centre of Trauma, Resilience and Growth, The University of Nottingham.

Saul Becker is Professor of Social Policy and Social Care and Head of the School of Sociology and Social Policy, The University of Nottingham.

Fiona Becker is a Senior Research Fellow in the School of Sociology and Social Policy, The University of Nottingham.

The right of Hannah Elwick, Stephen Joseph, Saul Becker and Fiona Becker to be identified as authors of this work has been asserted by them in accordance with the 1988 Copyright, Designs and Patents Act.

All rights reserved: no part of this publication may be reproduced, stored in a retrieval system, or transmitted in any forms or by any means, electronic, mechanical, photocopying, recording, or otherwise as part of research projects or surveys without specific written permission from the authors. The instrument in the Manual may be photocopied and used by practitioners within their own organisation for individual assessment or clinical use. Photocopies may not be made for distribution to other organisations. All other copyright restrictions remain in force.

Acknowledgements

The authors acknowledge with gratitude the help of The Princess Royal Trust Carers' Centres that piloted the Adult Carer Quality of Life questionnaire.

Funding

The development of the Adult Carer Quality of Life questionnaire and publication of the Manual was funded by The Princess Royal Trust for Carers.

Competing Interests

No financial or competing interests have been declared by the authors.

Download

This Manual can be downloaded for free from www.carers.org/professionals and www.saulbecker.co.uk

Citation

The Manual should be cited as: Elwick, H., Joseph, S., Becker, S. & Becker, F. (2010) *Manual for the Adult Carer Quality of Life Questionnaire* (AC-QoL). London: The Princess Royal Trust for Carers.

ISBN

13 9780853582748

Design and print by Touch Design

www.touchdesign.co.uk

Contents

		Page
1.	Context for the Development of the AC-QoL Questionnaire	04
1.1	Introduction	04
1.2	Recognising the needs of carers	04
2.	Using the Adult Carer Quality of Life Questionnaire	05
2.1	Introduction to the AC-QoL	05
2.2	Development of the AC-QoL	05
2.3	How to use the AC-QoL	06
2.4	Scoring of the AC-QoL	06
2.5	Interpretation of the scores	07
3.	References	07
	The Adult Carer Quality of Life Questionnaire (AC-QoL)	08
	Demographic Questions	10

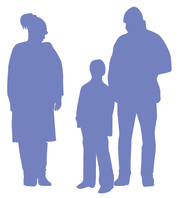

1. Context for the development of the AC-QoL Questionnaire

1.1 Introduction

The Adult Carer Quality of Life Questionnaire (AC-QoL) is a simple instrument for use with adult carers that measures quality of life in eight separate domains: support for caring; caring choice; caring stress; money matters; personal growth; sense of value; ability to care; and carer satisfaction. The questionnaire can be used on a single occasion in order to assess quality of life at the time of administration. In addition, the questionnaire is likely to be useful to assess the effectiveness of interventions. It can be easily administered before and after an intervention in order to evaluate whether the intervention has had an effect.

1.2 Recognising the needs of carers

Many adults care for family or friends. There are nearly six million people providing unpaid care in the UK. In the National Carer's Strategy (HM Government, 2008), it was reported that 71% of carers have had health problems which included poor physical and mental health. In turn poorer physical and mental health can affect the person's ability to care, with the majority of these carers reporting that their health problems also affected the person they were caring for.

Fulfilling a caring role can have a detrimental effect on the mental health of some carers (Carers UK, 2004). Carers have reported lower levels of self-esteem, a loss of self-confidence and greater psychological distress. Carers can also suffer from fatigue and stress, which can effect how they care and cope (Collins & Long, 2003). However, the negative impact of caring does not represent the total range of carers' experience (Brouwer, Exel, Berg, Bos & Koopmanschap, 2008). Carers often report that they have a sense of satisfaction, achievement, meaning and enjoyment from caring. Often this is through feeling appreciated for carrying out the caring role (Cohen, Colantonio & Vernich, 2002). Widening the range of how carers are affected to include both negative and positive aspects is important because research has suggested that a lack of positive impact in carers' quality of life can be indicative of problems in the caring role.

For example, a decrease or low sense of satisfaction as a carer can be a sign that there may be a potential breakdown in the caring relationship between the carer and the cared for individual (Archbold, Stewart, Greenlick & Harvath, 1992).

We do not include here a full review of the literature on the needs of carers or the outcomes of caring as there are useful sources elsewhere (see for example, Becker, 2008; Parker et al., 2010).

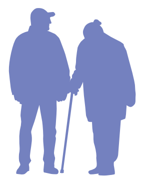

2. Using the Adult Carer Quality of Life Questionnaire

2.1 Introduction to the AC-QoL

The Adult Carer Quality of Life Questionnaire (AC-QoL) is a 40-item instrument that measures the overall quality of life for adult carers, and subscale scores for eight domains of quality of life:

1. Support for caring

This subscale measures the extent of support carers perceive that they receive; encompassing emotional, practical and professional support.

2. Caring choice

This subscale measures the extent to which carers feel that they have control over their own life, and are able to choose ventures outside caring, such as social activities.

3. Caring stress

This subscale measures the mental and physical stress from caring, such as exhaustion and depression.

4. Money matters

This subscale measures how carers feel about their financial situation.

5. Personal growth

This subscale measures how much the carer feels they have grown and developed, and the positive experience of the carers' circumstances.

6. Sense of value

This subscale measures the extent to which the carer feels they are valued and respected, and the positive relationship between the carer and the person they are caring for.

7. Ability to care

This subscale measures the extent to which the carer is able to provide care for the person they care for, how they cope with the caring role, and how they feel about their competency to care.

8. Carer satisfaction

This subscale measures the extent to which the carer is satisfied with their life and role as a carer, and how they feel about being a carer.

2.2 Development of the AC-QoL

Questionnaire items included in the AC-QoL were initially drawn up from a variety of sources and included: a review of the literature on carers; a review of scales used previously in carer research; an expert informed panel; and the involvement of carers. These processes allowed us to identify an extensive list of 100 questionnaire items which reflected all the factors that pertain to the quality of life for carers.

Three hundred and eighty-five adult unpaid carers between the ages of 19-93 were recruited from The Princess Royal Trust Carers' Centres in order to complete the pilot 100 item version of the questionnaire. Of these carers, 91 were male and 286 female (eight participants did not give their gender) and their average age was 63 years. The average length of time they had spent caring was 12 years, and the average amount of time spent caring per week was 114 hours per person. The purpose of the pilot was to ensure that the full range of questionnaire items were included and that the questionnaire items were easy to understand and answer by carers themselves.

The final step was to make the questionnaire items representative of the range of issues that are meaningful and relevant to adult carers, and to make it as simple as possible to administer and complete. Using statistical tests, the 100 item questionnaire was reduced to 40 items consisting of 8 subscales.

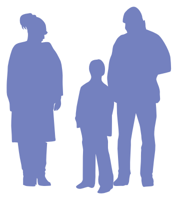

2.3 How to Use the AC-QoL

The AC-QoL is a self-report questionnaire that most carers will find straightforward to use. Carers should be invited to complete the questionnaire by themselves although some may require assistance. Carers should be given as much time as they need to fill in the questionnaire but on average it should take no longer than 10 minutes. Instructions on how to complete the questionnaire are given in the respondent information, and should be read before starting the questionnaire.

When completing the questionnaire, respondents should be given privacy and as much time as they need. It is important that respondents do not feel pressured to answer in a particular way. The completed questionnaires should be treated confidentially and remain anonymous whenever possible to ensure the carers' answers are a true indication of how they feel. Administration of the questionnaire should always be in line with the professional Code of Ethics appropriate for the organisation.

2.4 Scoring of the AC-QoL

In order to score the AC-QoL use the following scoring framework. Some of the questionnaire items are negatively worded and some are positively worded.

For the following questionnaire items:

1, 2, 3, 4, 5, 17, 18, 20, 21, 22, 23, 24, 25, 26, 27, 28, 29, 30, 31, 32, 33, 34, 35, 36, 39, & 40

Score:

Never	0
Some of the time	1
A lot of the time	2
Always	3

For the following questionnaire items:

6, 7, 8, 9, 10, 11, 12, 13, 14, 15, 16, 19, 37 & 38:

Score:

Never	3
Some of the time	2
A lot of the time	1
Always	0

Below is a scoring template. You may wish to make photocopies of this for use in scoring. Give each answer to a question a score of 0 to 3 using the scoring framework above. Then write it in the appropriate box. Add up each row for the score for each subscale, and add all the scores for the subscales to calculate the overall quality of life score.

Scoring Template for the Adult Carer Quality of Life Scale (AC-QoL)

Subscale	Questions					Subscale Total
Support for caring	Q1 ☐	Q2 ☐	Q3 ☐	Q4 ☐	Q5 ☐	☐
Caring choice	Q6 ☐	Q7 ☐	Q8 ☐	Q9 ☐	Q10 ☐	☐
Caring stress	Q11 ☐	Q12 ☐	Q13 ☐	Q14 ☐	Q15 ☐	☐
Money matters	Q16 ☐	Q17 ☐	Q18 ☐	Q19 ☐	Q20 ☐	☐
Personal growth	Q21 ☐	Q22 ☐	Q23 ☐	Q24 ☐	Q25 ☐	☐
Sense of value	Q26 ☐	Q27 ☐	Q28 ☐	Q29 ☐	Q30 ☐	☐
Ability to care	Q31 ☐	Q32 ☐	Q33 ☐	Q34 ☐	Q35 ☐	☐
Carer satisfaction	Q36 ☐	Q37 ☐	Q38 ☐	Q39 ☐	Q40 ☐	☐

Grand Total ☐

2.5 Interpretation of the Scores

Scores can be worked out for the total quality of life using the whole questionnaire, or for each subscale.

Scores on the overall questionnaire have a possible range of 0 to 120 with higher scores indicating greater quality of life.

0-40	Indicates a low reported quality of life, and may suggest problems or difficulties
41-80	Indicates a mid range reported quality of life
81+	Indicates a high reported quality of life

Scores on each of the eight subscales have a possible range of 0 to 15, with higher scores indicating greater quality of life on that subscale.

0-5	Indicates a low reported quality of life, and may suggest problems or difficulties
6-10	Indicates a mid range reported quality of life on that subscale
11+	Indicates a high reported quality of life on that subscale

3. References

Archbold, P. G., Stewart, B. J., Greenlick, M. R. & Harvath, T. A. (1992). *The clinical assessment of mutuality and preparedness in family caregivers to frail older people. Key aspects of elder care.* New York: Springer Publishing Company.

Becker, S. (2008). 'Informal family carers'. In K. Wilson, G. Ruch, M. Lymbery, & A. Cooper (eds.), *Social work: an introduction to contemporary practice.* London: Pearson Longman, pp. 431-460.

Brouwer, W. B. F., Exel, N., Berg, B., Bos, G. & Koopmanschap, M. (2008). Process utility from providing informal care: The benefit of caring. *Health Policy.* 74, 85-99.

Carers UK (2004). *In poor health: The impact of caring on health.* London: Carers UK.

Cohen, C. A., Colantonio, A. & Vernich, L. (2002). Positive aspects of caregiving: Rounding out the caregiving experience. *International Journal of Geriatric Psychiatry.* 17, 184-188.

Collins, S. & Long, A. (2003). Too tired to care? The psychological effects of working with trauma. *Journal of Psychiatric and Mental Health Nursing*, 10, 17–27.

HM Government (2008). *Carers at the heart of the 21st-century families and communities.* London: Department of Health.

Parker, G., Arksey, H. & Harden, M. (2010). *Scoping review on carers research.* York: Social Policy Research Unit.

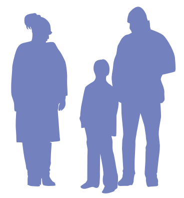

The Adult Carer Quality of Life Questionnaire (AC-QoL)

How to Fill in the Questionnaire

This questionnaire asks you about different aspects of your life as a carer. Please think about your experience as a carer within the last two weeks and please tick the box that applies next to each statement. There are no right or wrong answers; we are just interested in what life is like for you as a carer. The questionnaire shouldn't take more than 10 minutes.

Please answer all questions as honestly as you can.

Support for Caring

	Never	Some of the time	A lot of the time	Always
01. I have a good level of emotional support				
02. My needs as a carer are considered by professionals				
03. I am happy with the professional support that is provided to me				
04. I feel able to get the help and information I need				
05. I have all the practical support I need				

Caring Choice

	Never	Some of the time	A lot of the time	Always
06. I feel that my life is on hold because of caring				
07. My social life has suffered because of caring				
08. I feel I have less choice about my future due to caring				
09. I feel I have no control over my own life				
10. Caring stops me doing what I want to do				

Caring Stress

	Never	Some of the time	A lot of the time	Always
11. I feel depressed due to caring				
12. I feel worn out as a result of caring				
13. I am mentally exhausted by caring				
14. I am physically exhausted by caring				
15. I feel stressed as a result of caring				

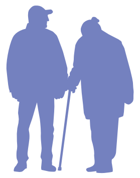

Money Matters

	Never	Some of the time	A lot of the time	Always
16. I worry about going into debt				
17. I feel satisfied with my financial situation				
18. I am able to save for a rainy day				
19. I worry about money				
20. There is enough money in our house to pay for the things we need				

Personal Growth

	Never	Some of the time	A lot of the time	Always
21. I have become a more tolerant person through my caring role				
22. Because of caring, I have learnt a lot about myself				
23. Because of caring, I feel that I have grown as a person				
24. I have experienced many positive things through caring				
25. I feel that I have become a better person by caring				

Sense of Value

	Never	Some of the time	A lot of the time	Always
26. I feel valued by the person I am looking after				
27. The person I look after respects me for what I do				
28. The person I look after makes me feel good about myself				
29. I get a lot from the person I am looking after				
30. I have a good relationship with the person I am caring for				

Ability to Care

	Never	Some of the time	A lot of the time	Always
31. I am satisfied with my performance as a carer				
32. I can take care of the needs of the person I am caring for				
33. I feel I am able to make the life of the person I am looking after better				
34. I can manage most situations with the person I care for				
35. I am able to deal with a difficult situation				

Carer Satisfaction

	Never	Some of the time	A lot of the time	Always
36. Caring is important to me				
37. I resent having to be a carer				
38. I feel frustrated with the person I am caring for				
39. I enjoy being a carer				
40. I am satisfied with my life as a carer				

Demographic Questions

A Please give your age ....

B Please indicate your gender (please tick) ☐ Male ☐ Female

C Please indicate your ethnicity (please tick one box)

- ☐ White - British
- ☐ White - Irish
- ☐ White - any other
- ☐ Mixed - White and Black Caribbean
- ☐ Mixed - White and Black African
- ☐ Mixed - White and Asian
- ☐ Mixed - Any other mixed background
- ☐ Asian/Asian British - Indian
- ☐ Asian/Asian British - Pakistani
- ☐ Asian/Asian British - Bangladeshi
- ☐ Asian/Asian British - Any other Asian
- ☐ Black/Black British - Caribbean
- ☐ Black/Black British - African
- ☐ Black/Black British - Any other
- ☐ Chinese
- ☐ Any other ethnic background

D How many hours do you spend caring per week? (please tick)

☐ 0-10 hours	☐ 11-20 hours	☐ 21-30 hours	☐ 31-40 hours
☐ 41-50 hours	☐ 51-60 hours	☐ 61-70 hours	☐ Greater than 71-hours

E How long have you been a carer for?

.... years

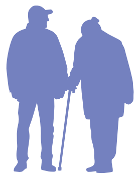

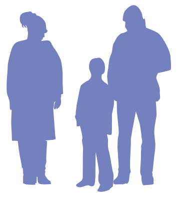

Supplement: Supplementary file 1 — Supplementary Material 1: Appendix: adult Carer Quality of Life Questionnaire [file 12955_2024_2317_MOESM1_ESM.pdf]
