# Supplementary material for: Predictors of quality of life among caregivers of patients with moderate to severe kidney disease: an Australian cross-sectional study
Source: Health Qual Life Outcomes. 2024 Dec 18;22:106. doi: 10.1186/s12955-024-02317-z (PMC11657763; doi:10.1186/s12955-024-02317-z)
Supplement: Supplementary file 2 — Supplementary Material 2: Table S1: Multiple logistic regression models showing factors associated with caregiver quality of life. [file 12955_2024_2317_MOESM2_ESM.docx]

**Supplementary Table 1**: Multiple logistic regression models showing factors associated with caregiver quality of life

| **Odds ratio (95% confidence interval)** | | | | | | | | | |
| --- | --- | --- | --- | --- | --- | --- | --- | --- | --- |
| **Characteristic** | **Support for Caring** | **Caring Choice** | **Caring Stress** | **Money Matters** | **Personal Growth** | **Sense of Value** | **Ability to Care** | **Carer Satisfaction** | **Overall scores** |
| **Care givers** |  |  |  |  |  |  |  |  |  |
| Age (Ref: <65 years) | 1 | 1 | 1 | 1 | 1 | 1 | 1 | 1 | 1 |
| ≥65 years | 2.2 (1.0-4.9) | 1.1 (0.5-2.4) | 1.4 (0.6-3.0) | 3.2 (1.3-7.6) **^*^** | 1.3 (0.6-3.0) | 1.5 (0.7-3.4) | 1.8 (0.8-3.9) | 1.0 (0.5-2.2) | 1.5 (0.7-3.3) |
| Gender (Ref: male) | 1 | 1 | 1 | 1 | 1 | 1 | 1 | 1 | 1 |
| Female | 2.7 (1.2-6.4) **^*^** | 1.3 (0.6-3.0) | 1.3 (0.6-3.0) | 1.0 (0.4-2.5) | 2.2 (0.9-5.4) | 2.8 (1.1-6.7) **^*^** | 0.8 (0.4-1.9) | 2.8 (1.2-6.5**) ^*^** | 1.5 (0.7-3.3) |
| Caring time (Ref: 0-30 hrs) | 1 | 1 | 1 | 1 | 1 | 1 | 1 | 1 | 1 |
| 31-60 hrs | 0.4 (0.1-1.2) | 0.5 (0.2-1.2) | 0.3 (0.1-0.7) **^**^** | 0.3 (0.1-1.2) | 0.9 (0.3-2.5) | 2.6 (0.9-7.3) | 3.1 (1.2-8.3**) ^*^** | 3.8 (1.4-10.7) **^*^** | 0.9 (0.3-2.2) |
| Over 61 hrs | 0.8 (0.4-1.6) | 0.5 (0.2-1.0) | 0.4 (0.2-0.9) **^*^** | 0.4 (0.2-0.9) **^*^** | 1.4 (0.7-2.9) | 2.2 (1.1-4.5) **^*^** | 2.7 (1.4-5.2) **^**^** | 2.0 (1.0-4.0) **^*^** | 0.7 (0.3-1.3) |
| Ethnicity (Ref: White) | 1 | 1 | 1 | 1 | 1 | 1 | 1 | 1 | 1 |
| Mixed | 0.9 (0.5-1.8) | 0.7 (0.4-1.3) | 0.9 (0.5-1.7) | 0.9 (0.4-1.9) | 2.1 (1.1-4.2) **^*^** | 0.9 (0.5-1.8) | 0.8 (0.4-1.6) | 1.1 (0.6-2.1) | 0.8 (0.4-1.5) |
| Other | 2.0 (0.5-8.2) | 1.3 (0.3-5.4) | 1.4 (0.3-5.7) | 1.2 (0.2-8.0) | 2.5 (0.6-10.7) | 0.7 (0.2-2.9) | 0.7 (0.2-2.8) | 0.8 (0.2-3.2) | 1.1 (0.3-4.5) |
| **Patients** |  |  |  |  |  |  |  |  |  |
| Age (Ref: <65 years) | 1 | 1 | 1 | 1 | 1 | 1 | 1 | 1 | 1 |
| ≥65 years | 1.1 (0.5-2.3) | 1.1 (0.5-2.3) | 0.8 (0.4-1.6) | 1.6 (0.7-3.8) | 1.0 (0.5-2.3) | 1.5 (0.7-3.3) | 1.2 (0.6-2.5) | 1.3 (0.6-2.7) | 1.1 (0.6-2.7) |
| Gender (Ref: male) | 1 | 1 | 1 | 1 | 1 | 1 | 1 | 1 | 1 |
| Female | 2.2 (0.9-5.1) | 1.6 (0.7-3.6) | 1.6 (0.7-3.6) | 1.2 (0.5-3.0) | 3.4 (1.4-8.1) **^**^** | 2.3 (1.0-5.3) | 1.5 (0.7-3.3) | 2.5 (1.1-5.8) **^*^** | 1.8 (0.8-4.1) |
| Stage of CKD (Ref: 3) | 1 | 1 | 1 | 1 | 1 | 1 | 1 | 1 | 1 |
| 4 | 1.5 (0.2-10.2) | 1.6 (0.3-9.8) | 0.7 (0.1-4.7) | 0.7 (0.1-4.6) | 1.6 (0.2-11.1) | 2.0 (0.3-14.3) | 0.8 (0.1-5.7) | 2.5 (0.4-14.8) | 2.2 (0.4-13.7) |
| 5 | 1.2 (0.2-7.5) | 1.0 (0.2-5.9) | 0.7 (0.1-4.6) | 0.7 (0.1-4.4) | 1.0 (0.2-6.4) | 0.8 (0.1-4.9) | 0.5 (0.1-3.0) | 1.0 (0.2-5.4) | 1.6 (0.3-9.3) |
| CCI (Ref: mild) | 1 | 1 | 1 | 1 | 1 | 1 | 1 | 1 | 1 |
| Moderate | 1.0 (0.4-2.5) | 1.9 (0.7-5.1) | 1.5 (0.6-4.0) | 1.4 (0.5-3.8) | 0.8 (0.3-2.1) | 0.4 (0.1-1.2) | 0.5 (0.2-1.2) | 0.9 (0.3-2.3) | 1.3 (0.5-3.3) |
| Severe | 0.6 (0.2-1.7) | 0.6 (0.2-1.8) | 0.6 (0.2-1.8) | 1.0 (0.3-3.0) | 1.0 (0.3-3.0) | 0.4 (0.1-1.2) | 0.5 (0.2-1.4) | 0.7 (0.2-2.0) | 0.7 (0.3-2.1) |
| Diabetes (Ref: No) | 1 | 1 | 1 | 1 | 1 | 1 | 1 | 1 | 1 |
| Yes | 1.3 (0.6-3.1) | 1.6 (0.7-3.8) | 1.6 (0.7-3.8) | 3.1 (1.2-7.9) **^*^** | 1.9 (0.8-5.6) | 2.0 (0.6-3.5) | 1.0 (0.4-2.3) | 1.6 (0.7-3.8) | 2.5 (1.1-5.7) **^*^** |
| SES (Ref: Upper) | 1 | 1 | 1 | 1 | 1 | 1 | 1 | 1 | 1 |
| Upper middle | 2.2 (0.8-6.0) | 1.7 (0.7-4.2) | 1.6 (0.6-4.1) | 0.6 (0.2-1.8) | 0.5 (0.2-1.3 | 0.8 (0.3-2.1) | 0.8 (0.3-2.1) | 0.5 (0.2-1.3) | 1.0 (0.4-2.4) |
| Lower middle | 0.8 (0.3-2.6) | 0.7 (0.2-1.9) | 1.0 (0.4-2.9) | 0.4 (0.1-1.5) | 1.0 (0.4-3.0) | 1.8 (0.6-5.5) | 1.0 (0.4-2.8) | 0.9 (0.3-2.5) | 0.7 (0.2-1.9) |
| Upper lower | 1.0 (0.3-2.5) | 0.5 (0.2-1.5) | 0.7 (0.2-2.0) | 0.6 (0.2-1.9) | 0.4 (0.1-1.1) | 0.9 (0.3-2.7) | 1.1 (0.4-3.1) | 0.5 (0.2-1.6) | 0.8 (0.3-2.4) |
| Lower | 4.2 (1.5-12.2) **^**^** | 1.9 (0.7-5.0) | 2.6 (1.0-7.2) | 1.3 (0.4-4.1) | 2.0 (0.7-5.6) | 2.0 (0.7-5.9) | 2.2 (0.8-6.2) | 1.3 (0.5-3.7) | 2.8 (1.0-7.7) **^*^** |

* p<0.05; **p<0.01; ***p<0.001; CKD-chronic kidney disease
